# Craniofacial Cephalometric Characteristics and Open Bite Deformity in Individuals with Amelogenesis Imperfecta—A Systematic Review and Meta-Analysis

**DOI:** 10.3390/jcm12113826

**Published:** 2023-06-02

**Authors:** Yassine Messaoudi, Stavros Kiliaridis, Gregory S. Antonarakis

**Affiliations:** 1 Division of Orthodontics, University Clinics of Dental Medicine, Faculty of Medicine, University of Geneva, 1 rue Michel-Servet, 1205 Geneva, Switzerland; stavros.kiliaridis@unige.ch (S.K.); gregory.antonarakis@unige.ch (G.S.A.); 2 Department of Orthodontics and Dentofacial Orthopedics, University of Bern, 3010 Bern, Switzerland

**Keywords:** amelogenesis imperfecta, orthodontics, open bite, hyperdivergent, vertical dimension, systematic review, meta-analysis, cephalometrics

## Abstract

Background: Individuals with amelogenesis imperfecta (AI) often present with malocclusions, especially a dental or skeletal anterior open bite (AOB). Objectives: To evaluate the craniofacial characteristics in individuals with AI. Material and methods: A systematic literature search was conducted with the PubMed, Web of Science, Embase and Google Scholar databases to identify studies relating to the cephalometric characteristics of individuals with AI, without any language or publication date restrictions. The grey literature was searched using Google Scholar, Opengrey and Worldcat. Only studies with a suitable control group for comparison were included. Data extraction and a risk of bias assessment were carried out. A meta-analysis was performed using the random effects model for cephalometric variables that were evaluated in at least three studies. Results: The initial literature search yielded 1857 articles. Following the removal of duplicates and a screening of the records, seven articles were included in the qualitative synthesis, representing a total of 242 individuals with AI. Four studies were included in the quantitative synthesis. The meta-analysis results showed that individuals with AI present a smaller SNB angle and larger ANB angle than those of control groups in the sagittal plane. In the vertical plane, those with AI present a smaller overbite and larger intermaxillary angle than those without AI. No statistically significant differences were found for the SNA angle when comparing the two groups. Conclusions: Individuals with AI seem to present with more vertical craniofacial growth, leading to an increased intermaxillary angle and decreased overbite. This possibly leads to a more retrognathic mandible with a larger ANB angle due to an anticipated posterior mandibular rotation.

## 1. Introduction

Amelogenesis imperfecta (AI) is a group of hereditary dental enamel disorders without evidence of generalized or systemic anomalies. It is considered to affect enamel formation of both the primary and the secondary dentition. AI has been associated with several other dental anomalies, including anterior open bite (AOB), eruption disturbances, congenitally missing teeth, root and crown resorption, pulpal calcifications and taurodontism [1].

Studies presenting the epidemiology of AI are scarce, and their data depend on the diagnostic criteria and the population studied, with the incidence of AI being reported to vary from 1 in 700 to 1 in 14,000, [2,3]. Family history, pedigree mapping and careful clinical observation are all used to diagnose AI. An accurate diagnosis of AI can be difficult due to heterogeneous phenotypes and the lack of a universally accepted nomenclature system.

The most widely used and accepted classification system seems to be that of Witkop [4], whereby AI can be classified into four major types based primarily on phenotype as follows: type I (hypoplastic) with well-mineralized but usually thin enamel and with deficiencies in quantity [5]; type II (hypomaturation) in which the thickness and hardness of the enamel is normal but it has an opaque mottled appearance and is vulnerable to wear due to a defect in the maturation process; type III (hypocalcified or hypomineralised) in which the enamel is normal in quantity and shape but is rough, soft and discoloured because it is poorly mineralized, resulting in soft enamel and severe tooth wear [6]; and type IV (hypomaturation/hypoplastic with taurodontism).

AI occurs in multiple inheritance patterns, including sex-linked, autosomal recessive, autosomal dominant and sporadic patterns. Enamel hypoplasia seems to be inherited predominantly as a sex-linked, incomplete, dominant trait and enamel hypomineralisation as an autosomal dominant trait [7,8]. AI has been associated with mutations in the amelogenin gene, AMELX, in families with an X-linked variation. Among individuals with AI, the reported distribution is roughly 60–70% with the hypoplastic type, 20–40% with the hypomaturation type and 7% with the hypocalcificied type [9].

Delayed eruption, the retention of teeth and frequent malocclusions have been reported in the hypomineralisation and hypoplasia AI types [10,11]. Persson and Sundell [12] and Bäckman and Adolfsson [13] compared measurements on cephalometric radiographs between individuals with AI and a control group, showing that those with AI present with cephalometric features characteristic of severe skeletal AOB. In some patients with AI, a narrow maxilla and reversed curve of Spee have been described. Anterior open bites are also commonly seen in patients with an increased gonial angle, facial height and mandibular plane angle [14]. 

The severity of the enamel phenotype does not seem to correspond, however, with the presence or severity of an anterior open bite [15]. Skeletal open bite can be observed in all types of AI but most frequently occurs in the hypocalcification type and less so in the hypoplastic type [16,17]. According to Rowley [17], skeletal open bite is totally absent in the hypomaturation type of AI. The prevalence of anterior open bite in individuals with AI varies between 24 and 60% [8,12,17,18], which is much higher than the 3–7% found in the general population [19].

AI is a rare disease and not all oral healthcare professionals are adequately trained to diagnose it correctly. Numerous case reports exist in the literature but only a few studies have dealt with the cephalometric characteristics of individuals with AI. Furthermore, there are many differences between these studies with regard to the populations and the cephalometric analyses carried out, and the rarity of AI makes it difficult to include large samples. The purpose of the present study was to identify craniofacial cephalometric characteristics in individuals with AI, in comparison to those without AI, using a meta-analysis methodology. Our aim was also to investigate whether any cephalometric differences were present between the different subtypes of AI. These investigations are important because they could help define appropriate treatment protocols for these patients and move towards establishing clinical guidelines.

## 2. Materials and Methods

A systematic review and meta-analysis were conducted according to predefined guidelines provided by the PRISMA group [20]. The research question formulated was based on the PICO model aiming to identify articles with the following characteristics: population (P)—patients with AI; intervention (I)—lateral cephalometry; comparison (C)—healthy control group without AI; and outcome (O)—lateral cephalometric characteristics. The protocol for the current systematic review and meta-analysis was not registered.

### 2.1. Search Strategy

The search strategies included a combination of controlled vocabulary and free terms, namely amelogenesis imperfecta AND (orthodontic* OR malocclusion OR occlusion OR cephalometric* OR open bite OR hyperdivergent* OR craniofacial). Databases searched were PubMed, Web of Science, Google Scholar and Embase to identify related studies. The grey literature was searched using Google Scholar, Opengrey and Worldcat. We manually searched the reference lists of included articles and publication lists of authors having worked in the field to identify any other relevant studies. Two investigators (YM and GSA) performed the literature search independently. The last update of the literature search was performed in April 2023.

### 2.2. Eligibility Criteria

Published studies were included with no restrictions based on language or date of publication. Inclusion criteria were as follows: studies with a control group, with a comparison to a group of patients with AI and with a minimum of 10 patients in each group. We excluded case reports, case series, studies without a suitable control group (less than 10 patients in each group) and studies with data that were incomplete or not available.

### 2.3. Study Selection and Data Collection

Two investigators (YM and GSA) assessed the articles and extracted data according to the predefined inclusion and exclusion criteria. Primary selection was based on the title and abstract. If data were insufficient, the full texts were read for clarification. All preselected articles were also downloaded as full-text articles and screened with the eligibility criteria in mind for final inclusion in this systematic review and meta-analysis. In case of disagreement between the two investigators, a third author (SK) was consulted.

Data extracted from each individual study included the following: country where the study took place, total number of patients, age of patients, number of control cases, number of AI cases, AI subtypes, sex distribution and cephalometric data (linear and angular). Corresponding authors were contacted in cases in which there were missing data or where clarifications were necessary.

### 2.4. Risk of Bias in Individual Studies

A qualitative assessment of the retrieved articles was conducted on the basis of the degree to which they exhibited well-established and accepted requirements for clinical research in this area. The qualitative criteria for this assessment were based on a previously used risk of bias assessment as defined by Flores-Mir et al. [21] and are as follows:

- Use of an appropriate control group (adequately matched for sex and age);
- Stated definitions of AI;
- Error of the method assessment with a measurement of examiner reliability;
- Blinding of the examiner(s);
- Definition of the cephalometric landmarks and angular and linear measurements used.

These five binary questions were evaluated. For each study, the number of “yes” answers were tallied, thereby determining the quality rating: 1–2 equal to “subpar”, 3–4 equal to “satisfactory” and 5 equal to “excellent”.

### 2.5. Statistical Analysis

Meta-analysis, using the random effects model, was performed to quantitatively evaluate the craniofacial characteristics in patients with AI, comparing them to a control group without AI. Cephalometric characteristics that were evaluated in a minimum of 3 studies were synthesised quantitatively. Specific meta-analysis software was used for this quantitative synthesis of included studies (Review Manager 5.4.1, RevMan, The Cochrane Collaboration, 2020), with the standard mean difference (SMD) as the output. Heterogeneity between studies was also assessed with the I^2^ statistic.

## 3. Results

### 3.1. Study Selection

**Figure 1:**
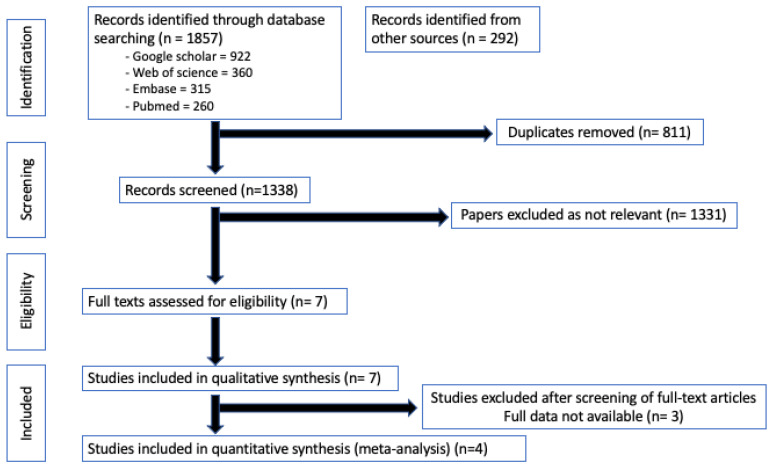
PRISMA flow diagram outlining the literature search and study selection for the qualitative and quantitative synthesis.

The initial literature search yielded a total of 1381 potentially eligible articles. A total of 447 duplicates were removed, with a total of 934 records being screened based on their title and abstract. Based on the previously defined inclusion and exclusion criteria, seven full texts were assessed for eligibility and were included in the qualitative synthesis. Only four of these articles met the criteria for inclusion in the quantitative synthesis [13,17,22,23] (Figure 1).

### 3.2. Study Characteristics

The seven articles included in the qualitative synthesis represented 242 individuals with AI (Table 1).

The age range was very heterogeneous with patients as young as three and as old as seventy included. The definition of the specific AI phenotype (subtype) was not available in all of the studies (Table 2). Based on the available data, five cephalometric characteristics were included in three or more studies and were examined in the quantitative synthesis, namely SNA, SNB, ANB, the intermaxillary angle and overbite (Table 2).

### 3.3. Risk of Bias

The qualitative criteria for this assessment were based on a previously used risk of bias assessment as defined by Flores-Mir et al. [21]. It was conducted on the included studies (Table 3). Six of the studies were judged to have a satisfactory risk of bias [12,13,15,23,24], while one study was judged to have subpar risk of bias [22].

### 3.4. Quantitative Synthesis of Results of Individual Studies

**Figure 2:**
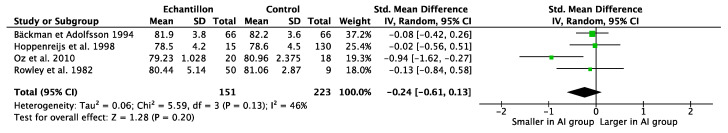
Forest plot comparing the SNA angle in individuals with amelogenesis imperfecta compared to a control group [13,17,22,23]. CI = confidence interval; IV = inverse variance; SD = standard deviation.

With regard to the SNA angle, an angular measurement, data were available in four studies involving 151 individuals with AI. A forest plot comparing the SNA angle in individuals with and without AI (Figure 2) shows no statistically significant difference between these groups, with moderate heterogeneity.

**Figure 3:**
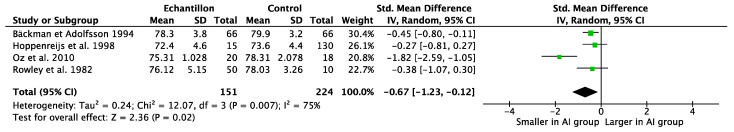
Forest plot comparing the SNB angle in individuals with amelogenesis imperfecta compared to a control group [13,17,22,23]. CI = confidence interval; IV = inverse variance; SD = standard deviation.

When looking at the SNB angle, an angular measurement, data were available in four studies involving 151 individuals with AI. A forest plot comparing the SNB angle in individuals with and without AI (Figure 3) shows a significant difference with a smaller SNB angle in those with AI (SMD = −0.67; 95% CI −1.23, −0.12; *p* = 0.02), with the studies showing considerable heterogeneity (I^2^ = 75%).

**Figure 4:**
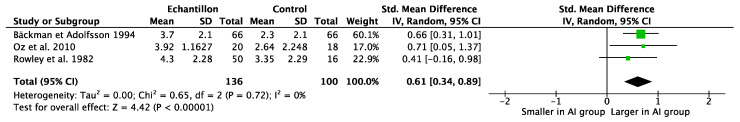
Forest plot comparing the ANB angle in individuals with amelogenesis imperfecta compared to a control group [13,17,23]. CI = confidence interval; IV = inverse variance; SD = standard deviation.

Data for the ANB angle, an angular measurement, were available from three studies representing 136 individuals with AI. A forest plot comparing the ANB angle between individuals with and without AI (Figure 4) shows a statistically significant larger ANB angle in those with AI (SMD = 0.61; 95% CI 0.34, 0.89; *p* < 0.01). Low heterogeneity was found among the studies (I^2^ = 0%).

**Figure 5:**
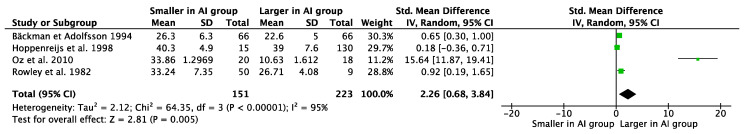
Forest plot comparing the intermaxillary angle in individuals with amelogenesis imperfecta compared to a control group [13,17,22,23]. CI = confidence interval; IV = inverse variance; SD = standard deviation.

The only vertical skeletal cephalometric characteristic that was included in three or more studies was the intermaxillary angle, for which data were available from four studies including 151 patients with AI. All of these studies defined the intermaxillary angle as the angle between the maxillary plane (anterior to the posterior nasal spine) and mandibular plane (Menton to Gonion). A forest plot comparing the intermaxillary angle between individuals with and without AI (Figure 5) shows a significantly larger intermaxillary angle in those with AI (SMD = 2.26; 95% CI 0.68, 3.84; *p* < 0.05), with considerable heterogeneity across studies (I^2^ = 95%). Because of this heterogeneity, sensitivity analyses were performed removing each individual study from the meta-analysis, and no significant changes to the overall mean difference were observed.

Nevertheless, the data from the study of Oz et al. [23] seemed to be an outlier with a mean intermaxillary angle in the control group of 10.63 degrees. This value, although extreme, was confirmed with the authors of the study who assured us that there were no errors in the data and this was indeed the mean intermaxillary angle of their sample.

**Figure 6:**
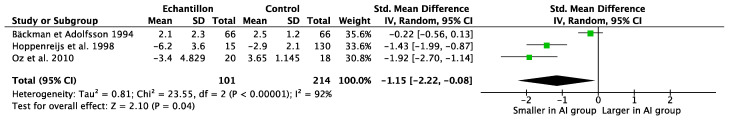
Forest plot comparing overbite in individuals with amelogenesis imperfecta compared to a control group [13,22,23]. CI = confidence interval; IV = inverse variance; SD = standard deviation.

With regard to dental cephalometric characteristics, only the overbite measurement, a linear measurement, was available from three of the included studies representing 101 individuals with AI. A forest plot comparing overbite in individuals with and without AI (Figure 6) showed that overbite was significantly smaller in those with AI (SMD = −1.15; 95% CI −2.22, −0.08; *p* = 0.04).

Considerable heterogeneity was identified across the included studies (I^2^ = 92%). Once again, due to a considerable amount of heterogeneity, a sensitivity analysis was performed. If the study of Hoppenreijs et al. [22] was excluded, the overall estimate changed with the difference between the two groups becoming statistically insignificant.

## 4. Discussion

The present meta-analysis results showed that individuals with AI, in the sagittal dimension, present a tendency towards a Class II skeletal sagittal relationship with a smaller SNB and a larger ANB than those of individuals without AI. In the vertical dimension, those with AI present with a more hyperdivergent tendency with a smaller overbite and a larger intermaxillary angle than those of individuals without AI. Individuals with AI seem to present with more vertical craniofacial morphologies with a possible posterior mandibular growth rotation leading to a larger intermaxillary angle, smaller overbite and a more retrognathic mandible with a larger ANB angle.

Only five cephalometric variables were investigated in three or more studies with presentations of means and standard deviations, and as a result, only these variables were analysed. It would have been interesting to look into variables such as the maxillary plane angle, the mandibular plane angle, the gonial angle and the tooth distance from the maxillary and mandibular planes, but data for these variables were not available from multiple studies. Unfortunately, data from Ravassipour et al. [15] were presented as z-scores and *p*-values and could therefore not be directly used in the meta-analysis. Data presented in the study of Persson and Sundell [12] did not include standard deviations and thus were also not usable in the present meta-analysis. Although the corresponding authors of these articles were contacted to obtain the missing data, we did not receive a response from Ravassipour et al. [15] or Persson and Sundell [12]. These two studies were thus excluded from the quantitative synthesis. Data from Pavlic et al. [24] only presented figures, and no values were available. These could not be obtained despite an attempt to contact the authors.

From the studies included, available data were used despite heterogeneity. The study of Hoppenreijs et al. [22] compared individuals with AI to individuals without AI but with open bite. This can therefore represent a certain bias in the data. Due to this inconsistency, however, sensitivity analyses were carried out. The study of Oz et al. [23] seemed to contain data that could be considered as an outlier, in which the intermaxillary angle of the control group had a mean value of 10.6 degrees. This value is largely inferior to the cephalometric norms. The authors of this study were contacted, they assured us that this was indeed the value that they found based on their data and this was therefore used as presented. Due to this inconsistency, however, sensitivity analyses were carried out. Moreover, as only the standard error was presented in Oz et al. [23], we calculated the standard deviation from their data using the mathematical formula ($SE=\frac{\sigma }{\surd n}$) where *SE* = standard error; *σ* = standard deviation; and *n* = sample size. In the study of Rowley et al. [17], the total number of patients included in the control group was not presented. They indicated that the results of two previous studies were used to provide normal cephalometric data with which to compare the study group. These two studies were those of Mills and Subtelny and Sakuda [25,26]. However, even after having consulted these studies’ authors, the question of the number of patients included in the control group was still not clarified. We decided therefore to use the formula for the *t*-test with two samples and two means to calculate the sample size (*n*) for the control group. The corresponding authors, Hoppenreijs et al. [22], Oz et al. [23] and Rowley et al. [17], were contacted to clarify these points for the quantitative data synthesis.

The results of the present meta-analysis suggest that individuals with AI seem to have more vertical craniofacial growth, but the pathophysiology leading to this growth and the AOB tendency remains unclear. There are two theories that can explain this association, namely, locally acting mechanisms due to environmental factors or a genetically determined skeletal growth pattern.

On the one hand, Witkop and Sauk [16] suggested that tongue interposition, probably provoked by an increased sensitivity of the teeth (secondary to the enamel disturbance), modifies the vertical alveolar growth through tooth eruption. In some studies, however, the association between AOB and tooth hypersensitivity has been questioned, since individuals with AI that report normal dental sensitivity still have a tendency for a similar skeletal pattern [17]. There are claims that an increased lower anterior facial height results in incompetent lips, and consequently, the tongue position may be altered, resulting in AOB [17]. Based on these statements, it can be said that the growth of the dentoalveolar complex may be altered by the tongue position, but it is questionable whether this could modify the morphology of the craniofacial complex to the extent that it was in the lateral cephalometric radiographic analyses [17].

Several studies have pointed out that in the general population, anterior open bite is frequently associated with a narrow maxilla, an open gonial angle, an inverted curve of Spee, the excessive eruption of posterior teeth and an increased lower face height [14,27]. The overeruption of the maxillary posterior teeth typically accompanies a skeletal hyperdivergent pattern, resulting in a downward and backward rotation of the mandible [15,17]. Given what we know about other diseases, despite the lack of direct evidence in individuals with AI, one can hypothesise that due to enamel defects, individuals with AI would be expected to have a decreased bite force that would favour the overeruption of the posterior teeth with the development of an anterior open bite.

On the other hand, embryological investigations suggest that enamel and the craniofacial skeleton may share a common ectomesenchymal origin [11]. In the study of Rowley et al. [17] conducted in the United Kingdom, 44% of individuals with AI presented vertical dysgnathia, defined as an increased intermaxillary angle (greater than 34°). Half of the patients in this group also had an anterior dental open bite. They suggested that the frequent association with AI and AOB could lead one to pinpoint a genetically determined craniofacial growth pattern, rather than local factors influencing dentoalveolar growth [17]. Cartwright et al. evaluated open bite characteristics in family members of individuals with AI that did not express enamel defects to differentiate whether the hyperdivergent skeletal phenotype was a familial trait independent of AI [28]. In some individuals with AI, skeletal hyperdivergency also occurred in family members with and without enamel defects, suggesting that these two phenotypic traits could be unrelated. While it is possible that the genetic mutations responsible for AI enamel defects predispose an individual to an anterior open bite, it also is possible that the AI-associated enamel defects and open bites are caused by different mechanisms.

The present systematic review and meta-analysis may present some limitations. A small number of studies were found in the literature and included, especially in the quantitative synthesis. AI is a rare disease, which may explain the relative lack of available studies. Furthermore, all of the included studies had either a moderate or high risk of bias, and no studies with a low risk of bias were included. The most common risk of bias was the heterogeneous data samples (age, type and origin of patients). This may, however, also be related the relative rareness of this condition. Finally, we noticed some concerns in the included studies with regard to unclear or missing data, but this was addressed to the best of our ability by contacting the authors and attempting to resolve these issues. Heterogeneity was albeit rather important for some of the studied variables.

There was unfortunately a lack of data segregated by the subtypes of AI, and this did not allow for an evaluation into the cephalometric differences between the different subtypes of AI as initially intended. Such information is important because it could help us define more appropriate diagnosis and condition-specific treatment protocols for these patients.

To date, there is no standardised procedure for the management of patients with AI and anterior open bite. Several therapeutic approaches can be considered, ranging from orthodontic treatments using appliances such as high-pull headgear, bite blocks, the multiloop edgewise archwire technique, skeletal anchorage or intrusion appliances [29,30] to conservative dentistry approaches and orthognathic surgery, with each approach presenting different advantages and disadvantages. The choice between a conservative or surgical treatment will depend on several factors, including one’s age, cooperation, the quantity and quality of their enamel, periodontal conditions and skeletal development [31]. A multidisciplinary specialist team including orthodontists, paediatric dentists, oral surgeons and prosthodontists is the key to successfully treating severe open bite problems in patients with AI. For adult patients with AI, the majority might need orthognathic surgery for definitive correction [17,22], but it is also known that the surgical correction of AOB is often prone to relapse [22]. Functional rehabilitation and retention are required in order to improve the patient’s chances of obtaining a result with an acceptable long-term level of stability.

Foumou-Moretti et al. [32] affirm that most published articles deal with the management of adult patients with AI and anterior open bite, with very few data concerning interceptive treatment in children. This highlights the need for studies dealing with younger individuals with AI, including an establishment of when the vertical problem begins to set in during growth. It would be interesting to evaluate children with AI at a young age to understand when these vertical problems are observed. It would also be interesting to look into factors such as the role of the masticatory muscles and occlusal forces on the development of anterior open bite in this population.

## 5. Conclusions

In conclusion, the present systematic review and meta-analysis evaluating craniofacial cephalometric characteristics in individuals with AI show that these patients seem to present with more vertical craniofacial growth, leading to an increased intermaxillary angle, decreased overbite and tendency towards a Class II sagittal skeletal pattern with a more retrognathic mandible. Despite the presence of these associations, one must keep in mind that causality cannot be confirmed. Given that AI is a rare disease, there is a desperate need for larger multi-centre well-designed studies to investigate craniofacial cephalometric characteristics in growing individuals with the different subtypes of AI.

## Figures and Tables

**Table 1:** Characteristics of included studies [12,13,15,17,22,23,24]. C = Control, AI = Amelogenesis imperfecta, M = Male, F = Female.

Study	Country	Age (Years) (Control/AI If Specified)	Total Number of Cases (Control + AI)	Number of Control Cases	Number of AI Cases	Number of Males (Total/AI If Specified)	Number of Females (Total/AI If Specified)
Bäckman et al., 1994 [13]	Sweden	AI: 6.8-21.2, mean 14.6	132	66	66	32/32	34/34
Hoppenreijs et al., 1998 [22]	Netherlands	C: 14.8-45.3 (mean 23.1) AI: 18.5-40.2 (mean 24)	145	130	15	28/AI 9	102/AI 6
Oz et al., 2010 [23]	Turkey	C: 11.83-16.17 (mean 13.13) AI: 9.25–15.5 (mean 12.25)	38	18	20	13/ AI 7	25/AI 13
Rowley et al., 1982 [17]	England	AI: 6.7–35, mean 18.1	66	16	50	Not specified/AI 14	Not specified/AI 36
Pavlic et al., 2010 [24]	Slovenia	AI: 6.5–15	14	3	11	7	6
Persson et al., 1982 [12]	Sweden	AI: 8–20	87	61	26	Not specified/AI 12	Not specified/AI 14
Ravassipour 2005 [15]	USA	Overall 3–70	88	34	54	48/Not specified	40/Not specified

**Table 2:** Characteristics of included studies (continued), [12,13,15,17,22,23,24]. C = Control, AI = Amelogenesis imperfecta, M = Male, F = Female.

Study	Outcomes Extracted	Type AI (Number of Patient in Each Group)
Bäckman et al., 1994 [13]	Intermaxillary angle (NL/ML)–SNA–SNB–ANB–Overbite	Hypoplastic (46) + Hypomineralization (18) + “Vertically ridged teeth” (2)
Hoppenreijs et al., 1998 [22]	Intermaxillary angle (PP-MP)–SNA–SNB–Overbite	Hypoplastic (6) + Hypomineralization (9)
Rowley et al., 1982 [17]	Intermaxillary angle (MMP angle)–SNA–SNB–ANB	Hypoplastic (18) + Hypocalcified (22) + Hypomaturation (10)
Oz et al., 2010 [23]	Intermaxillary angle (PP-MP)–SNA–SNB–ANB–Overbite	Not specified
Pavlic et al., 2010 [24]	Not included in quantitative metanalysis	Hypoplastic (11)
Persson et al., 1982 [12]	Not included in quantitative metanalysis	Hyposplastic (5 = 3M/2F) + Hypomineralisation (21)
Ravassipour 2005 [15]	Not included in quantitative metanalysis	Hypoplastic (5) + Hypocalcified (30) + Hypomaturation (2) + Not specified (17)

**Table 3:** Risk of bias of included studies [12,13,15,17,22,23,24].

Study	Control Group Matched for Age and Sex	Stated Definitions of AI	Measurement of Evaluator Reliability	Evaluator Masking Mentionned	Cephalometric Landmarks Clearly Defined	Overall Score
Bäckman et al., 1994 [13]	✓	✓	✓	✕	✓	Satisfactory
Hoppenreijs et al., 1998 [22]	✕	✓	✕	✕	✓	Subpar
Oz et al., 2010 [23]	✓	✓	✓	✕	✓	Satisfactory
Rowley et al., 1982 [17]	✕	✓	✓	✕	✓	Satisfactory
Persson et al., 1982 [12]	✓	✓	✕	✕	✓	Satisfactory
Ravassipour 2005 [15]	✕	✓	✓	✕	✓	Satisfactory
Pavlic et al., 2010 [24]	✕	✓	✓	✕	✓	Satisfactory

## Data Availability

The data underlying this article will be shared from the corresponding author upon reasonable request.
